# Ageing characterization data of lithium-ion battery with highly deteriorated state and wide range of state-of-health

**DOI:** 10.1016/j.dib.2021.107727

**Published:** 2021-12-16

**Authors:** Zhiyong Xia, Jaber A. Abu Qahouq

**Affiliations:** Department of Electrical and Computer Engineering, College of Engineering, University of Alabama, Tuscaloosa, AL 35487 United States

**Keywords:** Battery, Battery management system, Impedance, Lithium-ion, State-of-health

## Abstract

This paper presents ageing characterization data of two lithium-ion battery cells that have been put through a deep ageing process. At the end of ageing process, the values of state-of-health (SOH) of the battery cells drop down to around 15%. The battery cells are aged using a developed autonomous ageing platform which performs functions such as constant current (CC) discharging, CC charging, and constant voltage (CV) charging. Each time the battery cell completes 30 ageing cycles, battery performance tests including dc impedance measurement, minimum impedance measurement, and capacity calibration are conducted to characterize the ageing or health status of the battery cell. The collected battery dc impedance data, minimum impedance data, capacity data, and CC—CV charging time for the deeply aged battery cells are presented in this paper. The presented data has the potential to help in identifying battery ageing behavior patterns. It can also be utilized to investigate the correlation or relationship between different battery ageing characterization data and to develop SOH estimation methods for lithium-ion batteries with high degradation conditions, for examples, for second-use battery and when battery health exhibits unexpected faster deterioration.

## Nomenclature

**A. Acronyms**
CC: constant current
CV: constant voltage
SOC: state-of-charge
SOH: state-of-health

**B. Symbols**
*I*_Batt_: battery electric current in Ampere (A) unit
*Z*_dc_: battery impedance magnitude when the phase of complex impedance is equal to zero in Ohm (Ω) unit
*Z*_min_: battery minimum complex impedance magnitude in Ohm (Ω) unit
$Q_{avail}$: the actual available capacity or total amount of charges the battery can supply to a load (discharge) in Coulomb (C) unit
$Q_{nominal}$: nominal capacity of battery (i.e., the amount of charges the battery can supply to a load or discharge when it is new or not aged) in Coulomb (C) unit
$Q_{count}$: the amount of charges the battery discharges during a given time period from *t_0_* to *t_1_* in Coulomb (C) unit (Battery Coulomb counting result)
$t_{0}$: time instant $t_{0}$ in seconds unit
$t_{1}$: time instant $t_{1}$ in seconds unit
Δt: sampling time of battery electric current in seconds unit

## Specifications Table

**Table:** 

Subject	Electrical and Electronic Engineering
Specific subject area	Battery ageing characterization, battery impedance, and battery capacity
Type of data	Table Figure
How data were acquired	The battery dc impedance and battery minimum impedance are measured using Gamry Interface 5000E [1]. The battery capacity data and CC—CV charging time are measured and recorded using autonomous battery ageing platform which is described in [2].
Data format	Raw and analyzed
Parameters for data collection	Each time the battery cell completes 30 ageing cycles, the battery performance tests are conducted and CC—CV charging time is recorded. The dc impedance and minimum impedance values are measured at 100% state-of-charge (SOC), 60% SOC, and 20% SOC. The battery capacity is calibrated or calculated during the last ageing cycle of every 30 ageing cycles using Coulomb counting under CC discharging operation.
Description of data collection	CC—CV charging time and capacity data are collected using the developed battery autonomous ageing system. Battery dc impedance and minimum impedance values are recorded using Gamry Interface 5000E [1].
Data source location	Institution: The University of Alabama City/Town/Region: Tuscaloosa/Alabama Country: USA Latitude and longitude (and GPS coordinates, if possible) for collected samples/data: 33.21565034040115, −87.54360419954291
Data accessibility	Data is supplied with the article

## Value of the Data

• The presented data helps to characterize the ageing behavior patterns of lithium-ion battery over wide range of degradation levels down to around 15% state-of-health (deeply aged with high degradation condition). The ageing characterization data of highly deteriorated lithium-ion battery is not publicly available and therefore the presented data fills a currently existing gap in the publicly available data.
• Researchers who have interests in battery management system and battery health estimation can utilize the provided data in this paper to further investigate battery ageing characterization and the development of SOH estimation methods especially for wide range of health conditions.
• The presented data can be further utilized to investigate the correlation or relationship between different battery ageing characterization data, to develop SOH estimation methods for lithium-ion batteries with high degradation conditions, for examples, for second-use battery and when battery health exhibits unexpected faster deterioration, and to help understand the ageing evolving trend of battery.

## 1 Data Description

**Fig. 1:**
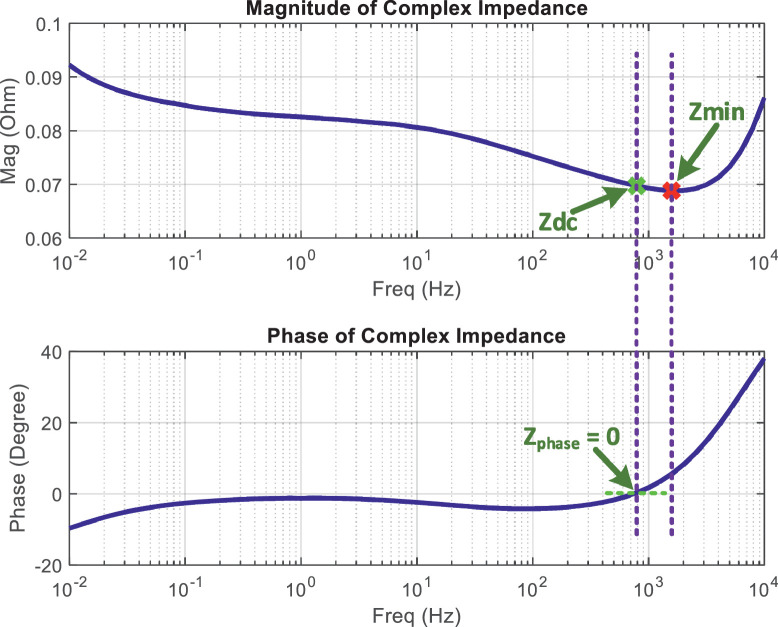
The illustration for the definition of *Z*_dc_ and *Z*_min_.

The ageing characterization data for two lithium-ion battery cells is collected as the battery health (or capacity) decreases down to around 15%. The collected battery ageing characterization data at different ageing cycles is listed in Tables 1 and 2. The battery ageing characterization data includes battery dc impedance (*Z*_dc_), battery minimum impedance (*Z*_min_), CC charging time, CV charging time, total charging time (CC charging time + CV charging time), and SOH values. Fig. 1 illustrates the definition of *Z*_dc_ and *Z*_min_. *Z*_dc_ refers to the magnitude of battery impedance when the phase of battery complex impedance is equal to zero. *Z*_min_ refers to the minimum magnitude value of the battery complex impedance within the frequency range from 0.01 Hz to 10 kHz. Over the ageing process, *Z*_dc_ and *Z*_min_ values are measured and recorded at different SOC conditions, i.e., 100% SOC, 60% SOC, and 20% SOC. The SOH values are calculated using Coulomb counting method, which is discussed in the next section of this paper (i.e., Experimental Design, Materials and Methods section). At the end of ageing process, i.e., when the ageing cycle is 630 in this paper, the battery cells are highly deteriorated with SOH value of 15.52% for battery #1 and SOH value of 12.4% for battery #2. Compared with common publicly available battery ageing datasets where battery cells are aged only up to approximately 70%–80% SOH [3], [4], [5], [6], [7], the battery ageing characterization dataset in this paper covers wide range of SOH down to around 15%. Therefore, the dataset presented in this paper can be utilized to investigate the correlation or relationship between different battery ageing characterization data and to develop SOH estimation methods for lithium-ion batteries that are deeply aged with high degradation conditions, for examples, for second-use and when battery health exhibits unexpected faster deterioration [8].

**Table 1:** Battery #1 ageing characterization data at different ageing cycles.

Cycle	Z_dc_@100% SOC (Ω)	Z_dc_@60% SOC (Ω)	Z_dc_@20% SOC (Ω)	Z_min_@100% SOC (Ω)	Z_min_@60% SOC (Ω)	Z_min_@20% SOC (Ω)	CC charge time (minutes)	CV charge time (minutes)	Total charge time (minutes)	SOH (%)
1	0.069733	0.06959	0.069624	0.068831	0.068709	0.068771	31.95	126.51	158.46	94.94
30	0.069729	0.069635	0.069631	0.068835	0.068751	0.069042	30.79	114.85	145.64	93.34
60	0.06988	0.069842	0.069713	0.068996	0.06894	0.069611	23.86	108.49	132.35	89.73
90	0.070795	0.070927	0.070634	0.069904	0.069983	0.070921	23.39	102.19	125.59	80.92
120	0.071607	0.071849	0.071552	0.070693	0.070882	0.071687	22.14	102.02	124.17	76.19
150	0.072111	0.072414	0.072137	0.071379	0.071436	0.072125	20.64	94.35	115.00	73.76
180	0.072617	0.072979	0.072734	0.071667	0.071991	0.072557	17.44	92.39	109.83	71.56
210	0.073279	0.073714	0.073523	0.072302	0.072713	0.073121	16.42	89.14	105.57	68.96
240	0.073828	0.074322	0.074183	0.073288	0.073311	0.073595	16.31	91.31	107.62	66.98
270	0.074544	0.075111	0.07505	0.073514	0.074089	0.074227	15.89	91.78	107.68	64.59
300	0.075668	0.076348	0.076423	0.07459	0.07531	0.075259	15.02	92.72	107.74	61.17
330	0.076019	0.077186	0.077714	0.075016	0.076133	0.076362	12.93	93.68	106.62	59.06
360	0.078239	0.078852	0.079071	0.076955	0.077454	0.077692	11.33	94.69	106.02	55.17
390	0.079037	0.081234	0.080756	0.077679	0.079887	0.079546	10.13	95.46	105.59	51.58
420	0.080462	0.080538	0.081688	0.079031	0.079491	0.080491	8.84	94.30	103.15	49.49
450	0.081474	0.082559	0.083034	0.080301	0.081296	0.081685	7.42	95.43	102.86	47.30
480	0.081558	0.083762	0.08467	0.080373	0.08254	0.083264	7.19	94.21	101.41	46.32
510	0.083566	0.083551	0.084077	0.082189	0.081972	0.082793	7.01	95.61	102.62	46.14
540	0.083372	0.085543	0.087031	0.081787	0.084198	0.085454	6.64	88.32	94.96	41.99
570	0.088103	0.091988	0.090913	0.086577	0.090521	0.089566	3.03	83.22	86.25	34.98
600	0.089273	0.095729	0.094826	0.087488	0.093726	0.092885	2.12	81.68	83.80	28.46
630	0.103558	0.102825	0.104732	0.101762	0.100861	0.102449	0.61	67.31	67.92	15.52

**Table 2:** Battery #2 ageing characterization data at different ageing cycles.

Cycle	Z_dc_@100% SOC (Ω)	Z_dc_@60% SOC (Ω)	Z_dc_@20% SOC (Ω)	Z_min_@100% SOC (Ω)	Z_min_@60% SOC (Ω)	Z_min_@20% SOC (Ω)	CC charge time (minutes)	CV charge time (minutes)	Total charge time (minutes)	SOH (%)
1	0.066665	0.067224	0.067258	0.065802	0.066373	0.066003	32.90	132.77	165.68	93.79
30	0.067497	0.067267	0.067523	0.066632	0.066513	0.066011	31.09	115.66	146.75	89.73
60	0.067644	0.067781	0.06808	0.066788	0.066907	0.066088	25.13	111.00	136.13	84.82
90	0.06853	0.068834	0.068509	0.067667	0.067918	0.068233	22.60	109.06	131.67	75.39
120	0.069817	0.069729	0.069608	0.068925	0.068791	0.069477	22.35	99.24	121.59	70.33
150	0.070308	0.071111	0.070045	0.069594	0.07015	0.070033	20.86	98.36	119.22	69.35
180	0.070802	0.071666	0.070625	0.069875	0.070695	0.070453	17.76	98.27	116.03	66.27
210	0.071447	0.072387	0.071391	0.070495	0.071404	0.071001	17.36	97.80	115.16	65.13
240	0.072794	0.072984	0.072328	0.072262	0.071991	0.071755	15.77	96.46	112.23	64.72
270	0.0735	0.07406	0.073173	0.072485	0.073052	0.072371	14.64	96.33	110.97	57.95
300	0.074609	0.075279	0.074894	0.073546	0.074255	0.073754	14.17	94.94	109.12	57.64
330	0.074954	0.076106	0.07616	0.073965	0.075067	0.074835	13.22	94.20	107.42	52.60
360	0.077144	0.077748	0.077489	0.075878	0.076369	0.076138	11.40	89.65	101.06	52.31
390	0.078247	0.080146	0.079141	0.076902	0.078816	0.077955	9.60	89.48	99.08	48.85
420	0.079657	0.079459	0.080218	0.07824	0.078426	0.079042	8.76	89.13	97.89	48.76
450	0.08066	0.081452	0.081539	0.079498	0.080206	0.080215	8.37	88.55	96.93	45.17
480	0.080334	0.08264	0.083146	0.079168	0.081434	0.081765	7.15	88.41	95.56	40.97
510	0.082312	0.082632	0.082984	0.080956	0.08107	0.080717	5.54	86.81	92.36	40.27
540	0.082122	0.084602	0.085899	0.08056	0.083272	0.084343	5.00	85.41	90.41	39.58
570	0.086781	0.090976	0.089731	0.085278	0.089525	0.088402	4.62	83.25	87.88	28.69
600	0.087934	0.094675	0.093878	0.086175	0.092695	0.091956	2.14	79.05	81.20	27.75
630	0.102005	0.10118	0.103495	0.100235	0.099248	0.102327	0.68	64.33	65.02	12.40

**Fig. 2:**
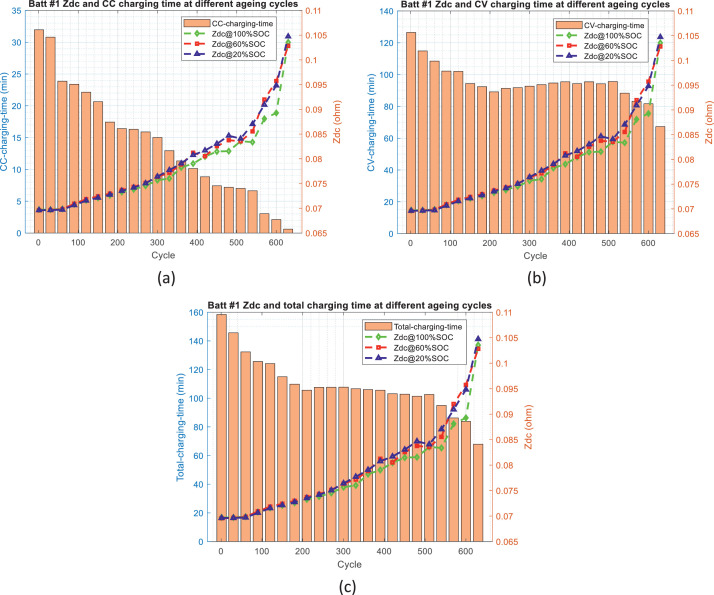
The distribution of *Z*_dc_ and charging time over the ageing process for battery #1: (a) *Z*_dc_ and CC charging time, (b) *Z*_dc_ and CV charging time, and (c) *Z*_dc_ and total charging time.

**Fig. 3:**
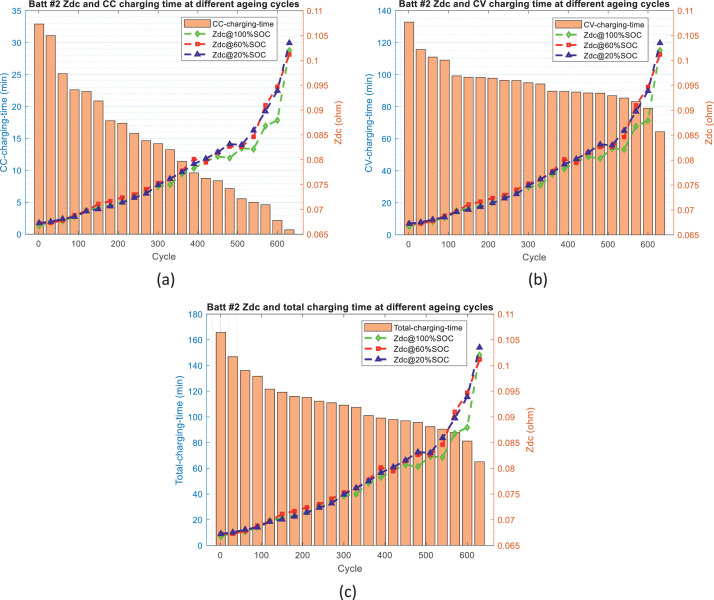
The distribution of *Z*_dc_ and charging time over the ageing process for battery #2: (a) *Z*_dc_ and CC charging time, (b) *Z*_dc_ and CV charging time, and (c) *Z*_dc_ and total charging time.

**Fig. 4:**
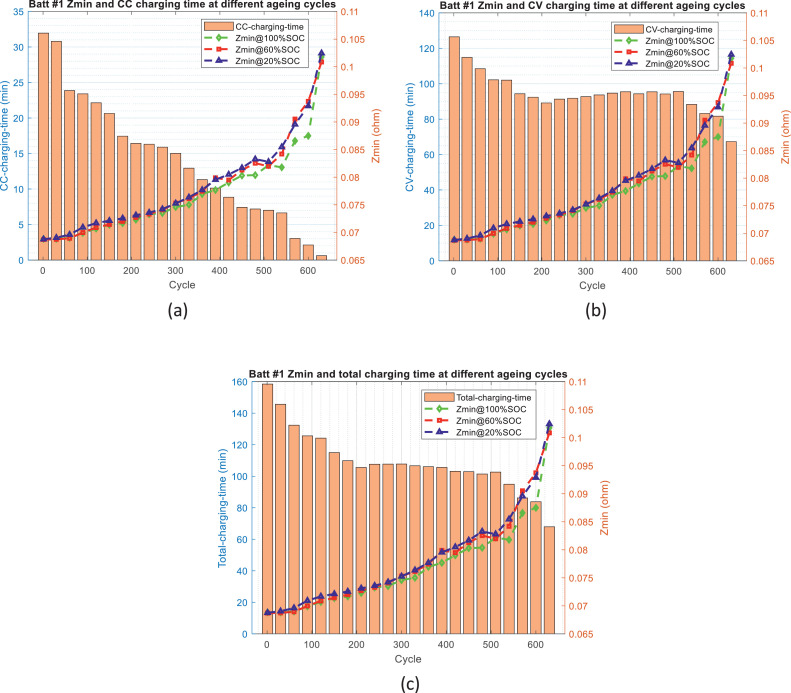
The distribution of *Z*_min_ and charging time over ageing process for battery #1: (a) *Z*_min_ and CC charging time, (b) *Z*_min_ and CV charging time, and (c) *Z*_min_ and total charging time.

**Fig. 5:**
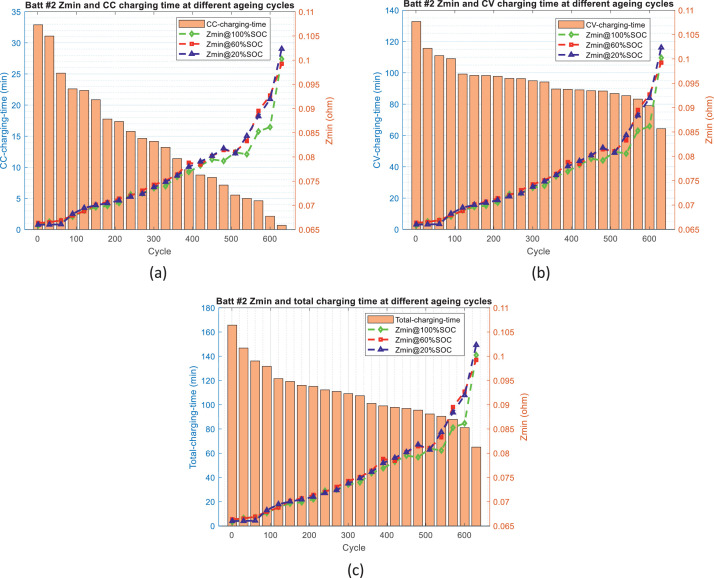
The distribution of *Z*_min_ and charging time over ageing process for battery #2: (a) *Z*_min_ and CC charging time, (b) *Z*_min_ and CV charging time, and (c) *Z*_min_ and total charging time.

Figs. 2–5 show the distribution of battery impedance (*Z*_dc_ and *Z*_min_) and battery charging time (CC charging time, CV charging time, and total charging time) over the ageing process. These figures can be utilized to identify or investigate the correlation between battery impedance and battery charging time over the ageing process.

**Fig. 6:**
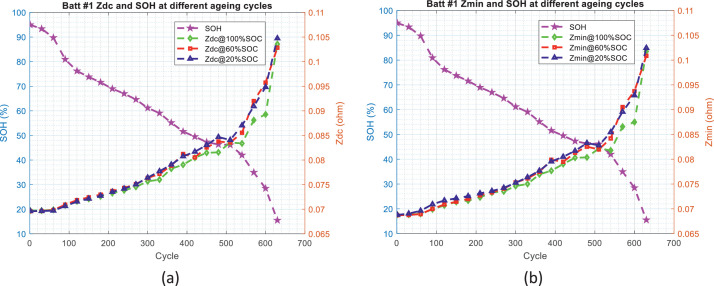
The distribution of battery impedance value and SOH values over the ageing process for battery #1: (a) *Z*_dc_ and SOH values and (b) *Z*_min_ and SOH values.

**Fig. 7:**
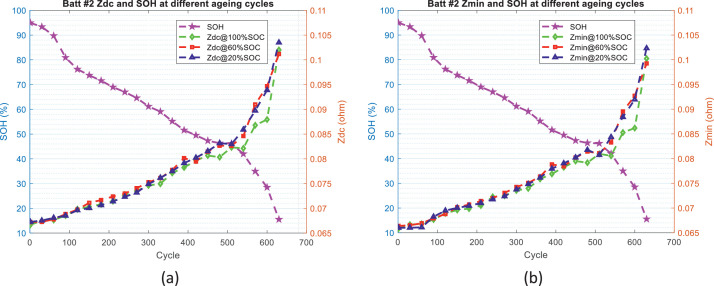
The distribution of battery impedance value and SOH values over the ageing process for battery #2: (a) *Z*_dc_ and SOH values and (b) *Z*_min_ and SOH values.

**Fig. 8:**
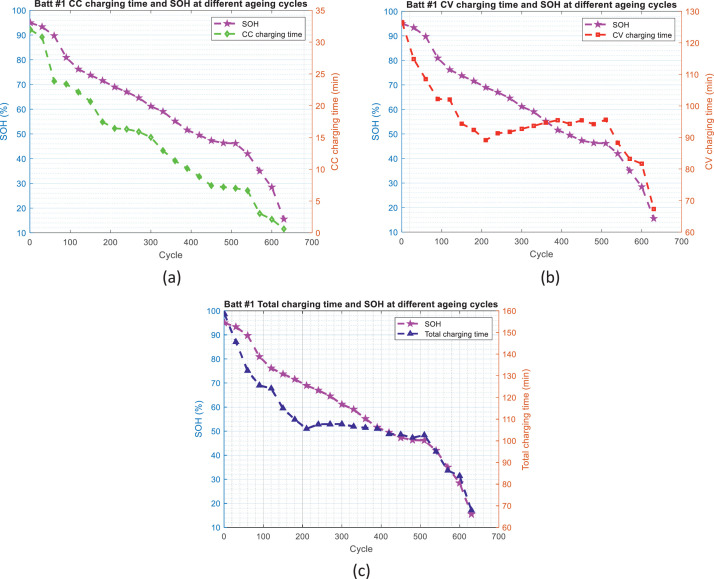
The distribution of battery charging time and SOH values over the ageing process for battery #1: (a) CC charging time and SOH values, (b) CV charging time and SOH values, and (c) total charging time and SOH values.

**Fig. 9:**
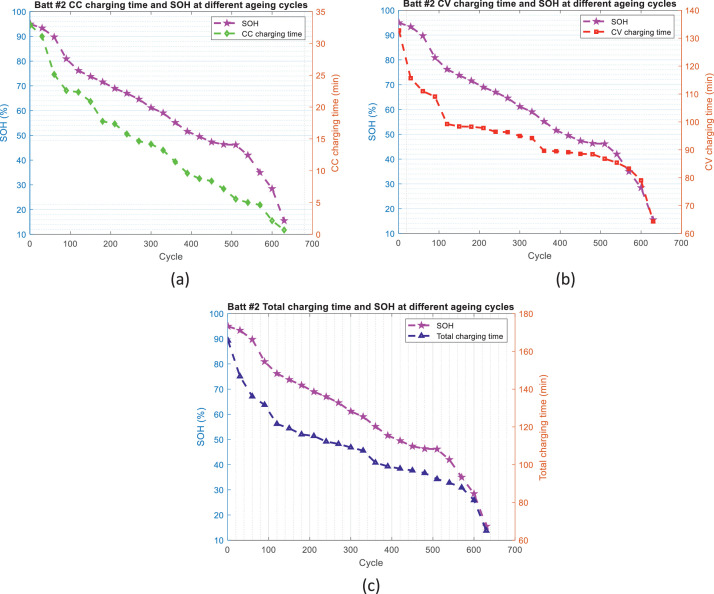
The distribution of battery charging time and SOH values over the ageing process for battery #2: (a) CC charging time and SOH values, (b) CV charging time and SOH values, and (c) total charging time and SOH values.

In order to develop SOH estimators for the battery, the first step is to identify the features or indicators which can reflect the health status of the battery or have relevant correlation with SOH values. For this purpose, Figs. 6 and 7 illustrate the evolution trend of the battery impedance (*Z*_dc_ and *Z*_min_) and SOH values, and Figs. 8 and 9 illustrate the evolution trend of the battery charging time (CC charging time, CV charging time, and total charging time) and SOH values.

Battery cells from different manufacturers may exhibit different battery ageing characteristics [9]. One of the reasons for this is because of the different chemistry compositions of different battery cells. However, the ageing effects on the presented parameters are expected to be same for the same battery type. The data presented in this paper is mainly used to reflect the ageing characteristics of the selected battery type from one manufacturer.

## 2 Experimental Design, Materials and Methods

The model of the utilized lithium-ion battery cell in the conducted ageing experiment is Tenergy ICR 16,650–2600 [10]. The main manufacturer datasheet parameters of interest of this type of battery include: nominal capacity is 2.6 Ah, initial impedance at 1 kHz is less than 65 mΩ, and average working voltage is 3.7 V [10].

The definition of SOH which is used in this paper is based on the concept of capacity fading [7], and it can be calculated using Eq. (1).

(1) $$SOH=\frac{Q_{avail}}{Q_{nominal}}=1-\frac{Q_{nominal}-Q_{avail}}{Q_{nominal}}=1-capacity\;fading$$

Where $Q_{avail}$ is the actual available capacity or total amount of charges the battery can supply to a load (discharge), which can be obtained using Coulomb counting method, and $Q_{nominal}$ is the nominal capacity of battery, which can be obtained from the documents of the manufacturer. Coulomb counting method is used to count the amount of charges the battery cell discharges based on Eq. (2) [11].

(2) $$Q_{count}=\int_{t_{0}}^{t_{1}}I_{Batt}dt=\sum_{t_{0}}^{t_{1}}\left(I_{Batt}\times \;\Delta t\right)$$

Where $Q_{count}$ is the amount of charges the battery cell discharges during time period from *t_0_* to *t_1_*, i.e., the Coulomb counting result, *I_Batt_* is the battery electric current measured value during the time period Δt, and Δt is the sampling time (10 µs in this paper) of battery electric current. Capacity fading is another variable used to indicate the degradation level of battery cell, and it can be calculated based on Eq. (3). The range of the value of capacity fading is from 0 to 1 (or 0% to 100%). The larger the value of capacity fading is, the more deteriorated the health of the battery is (i.e., the lower SOH is).

(3) $$capacity\;fading=\frac{Q_{nominal}-Q_{avail}}{Q_{nominal}}$$

The range of SOH value is from 0 to 1 (or 0% to 100%). The smaller the value of SOH is, the more deteriorated the battery is.

As an important performance characterization parameter of battery, battery impedance can be utilized to study and monitor the battery operation status and to estimate other internal state variables of the battery (for examples SOC, SOH, and battery internal temperature) [12], [13], [14], [15]. The values of *Z*_dc_ and *Z*_min_ are two important characteristic parameters of the battery impedance [7,[12], [13], [14], [15], and they are utilized as two ageing characterization parameters in this paper. During the ageing process of the lithium-ion battery in this paper, *Z*_dc_ and *Z*_min_ are measured. Battery charging time is also utilized to reflect the operation or health status of the battery [4], [5], [6]. Therefore, battery charging time is also measured in the conducted ageing experiment in this paper to characterize the ageing or performance of battery cell. The collected data for the battery charging time in this paper includes CC charging time, CV charging time, and total charging time.

**Fig. 10:**
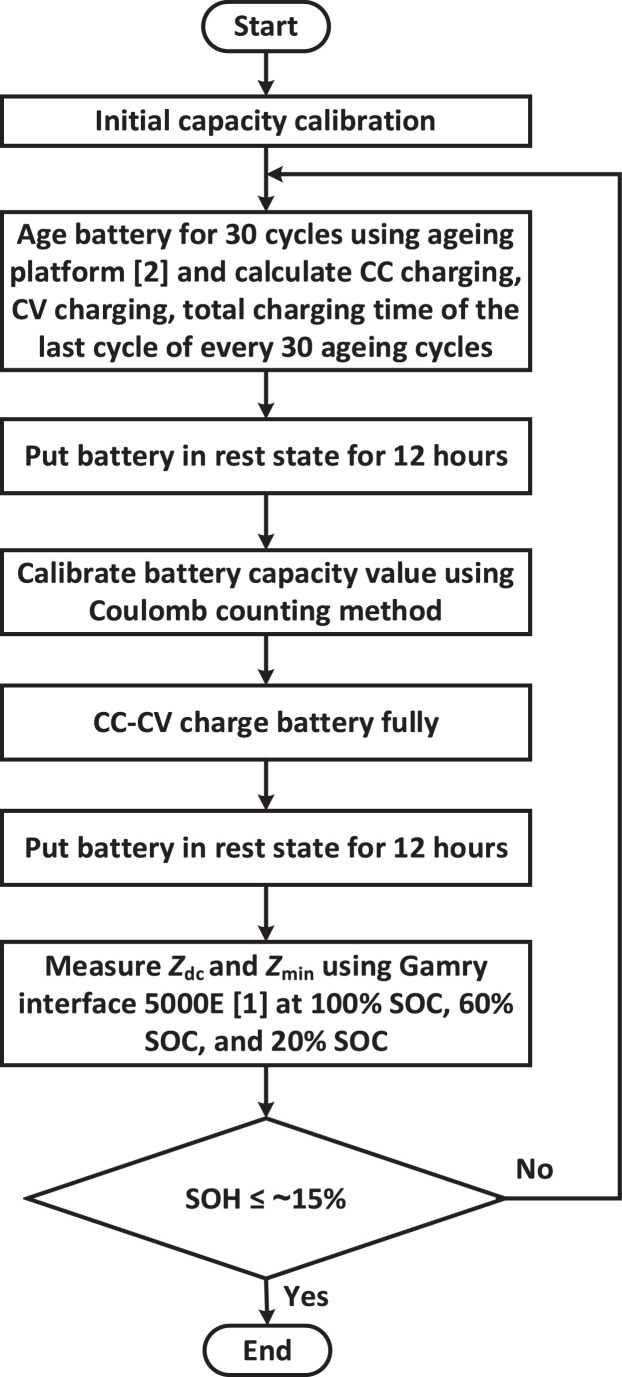
The flowchart of battery ageing and performance testing procedure utilized to measure and collect the presented data in this paper.

Fig. 10 shows the utilized ageing and performance testing procedure used to obtain the presented data in this paper. Before conducting the ageing experiment, the initial battery capacity is calibrated using Coulomb counting method.

After the initial capacity calibration, the battery cell is subjected to 30 ageing cycles using the developed autonomous ageing platform. The details about this ageing platform can be found in [2]. During each ageing cycle, the battery cell goes through one full CC discharging operation and one complete CC—CV charging operation. The CC discharging current is set to be 1 C (i.e., 2.6 A) during the ageing process. The discharging operation is terminated when the battery voltage drops down to 2.7 V. The CC charging current is also set to be 1 C (i.e., 2.6 A) during the ageing process. The CC charging operation is terminated when the battery voltage reaches 4.2 V. After the CC charging operation, CV charging operation is initiated to charge the battery until the battery current decreases down to 0.05 A (which is the condition to end the CV charging operation). For every 30 ageing cycles, CC charging time, CV charging time, and total charging time for the last ageing cycle are recorded by the developed ageing platform.

After completing 30 ageing cycles, the battery cell is put in rest state for 12 hours in order to achieve thermal equilibrium before calibrating the available capacity of the battery. This can help to obtain accurate capacity calibration results. After the capacity calibration, the battery is fully charged again to 100% SOC using CC—CV charging method and then the battery cell is put in rest state again for 12 hours. In the next step, Gamry interface 5000E [1] equipment is used to measure *Z*_dc_ and *Z*_min_ at 100% SOC, 60% SOC, and 20% SOC. CC discharging is utilized to discharge the battery cell from one SOC value to another SOC value, e.g., from 100% SOC to 60% SOC. After each discharging operation and before measuring battery impedance at one specific SOC value, the battery cell is always put in rest state for 12 hours to reach the thermal equilibrium state. The ageing process and battery performance tests are conducted until the SOH value of battery cell decreases to around 15%.

## CRediT authorship contribution statement

**Zhiyong Xia:** Investigation, Methodology, Data curation, Visualization, Writing – original draft. **Jaber A. Abu Qahouq:** Funding acquisition, Project administration, Investigation, Methodology, Visualization, Supervision, Writing – review & editing.

## Declaration of Competing Interest

The authors declare that they have no known competing financial interests or personal relationships which have or could be perceived to have influenced the work reported in this article.
